# Trends in US public confidence in science and opportunities for progress

**DOI:** 10.1073/pnas.2319488121

**Published:** 2024-03-04

**Authors:** Arthur Lupia, David B. Allison, Kathleen Hall Jamieson, Jennifer Heimberg, Magdalena Skipper, Susan M. Wolf

**Affiliations:** ^a^Office of the Vice President for Research and Department of Political Science, University of Michigan, Ann Arbor, MI 48109; ^b^Dean, School of Public Health, Indiana University, Bloomington, IN 47405; ^c^Annenberg Public Policy Center, University of Pennsylvania, Philadelphia, PA 19104; ^d^Policy and Global Affairs Division, The National Academies of Sciences, Engineering, and Medicine, Washington, DC 20001; ^e^Nature, London N1 9XW, United Kingdom; ^f^University of Minnesota Law School and Medical School, Minneapolis, MN 55455

## Abstract

In recent years, many questions have been raised about whether public confidence in science is changing. To clarify recent trends in the public’s confidence and factors that are associated with these feelings, an effort initiated by the National Academies’ Strategic Council for Research Excellence, Integrity, and Trust (the Strategic Council) analyzed findings from multiple survey research organizations. The Strategic Council’s effort, which began in 2022, found that U.S. public confidence in science, the scientific community, and leaders of scientific communities is high relative to other civic, cultural, and governmental institutions for which researchers regularly collect such data. However, confidence in these institutions has fallen during the previous 5 years. Science’s decline, while real, is similar to or less than that in the other groups. A recent study goes into greater detail by exploring public views of science. From these data, we observe that many of the surveyed U.S. public question the extent to which scientists share their values or overcome personal biases when presenting conclusions. At the same time, large majorities agree on certain types of actions that they want scientists to take. For example, 84% respond that it is “somewhat important” or “very important” for scientists to disclose their funders. Ninety-two percent (92%) offer the same responses to scientists “being open to changing their minds based on new evidence.” Collectively, these data clarify how the U.S. public views science and scientists. They also suggest actions that can affect public confidence in science and scientists in the years to come.

Science’s capacity to produce discoveries that help people better understand critical aspects of their universe, the environments in which they live, and one another is undisputed. Science is at the core of transformative technologies and innovative new materials and practices that improve health, increase economic opportunity, and enhance the quality of life for people around the world. Some scientific pursuits also provoke societal controversy. Topics such as climate change and vaccine safety not only spark debate but also lead some people to question the integrity of the science itself.

Phenomena such as these yield headlines such as “Can the public’s trust in science—and scientists—be restored?” (1). Indeed, in recent years, media, individuals, and scientific organizations have expressed a range of opinions about whether public confidence in science is declining and have offered conjecture and evidence about factors underlying public confidence in science (2, 3) and affecting support for funding it (4). The National Academies’ Strategic Council for Research Excellence, Integrity, and Trust (the Strategic Council) initiated this study to examine both public confidence and the factors affecting it.

The Strategic Council was formed in 2021 (5). Its initial activities have included examining ways: to make potential conflicts of interest easier to identify and disclose, to improve incentives associated with correcting the scientific record when mistakes are found (that is, improving the retraction process), to evaluate frameworks to increase incentives for scientific integrity, to explore how research laboratories and larger scientific groups can more effectively integrate scientific integrity and research ethics into their scientific practice, and to assess public confidence in science (the purpose of this article). In general, the Strategic Council is seeking ways to work with a wide range of partners to support scientific excellence while promoting practices that strengthen integrity and reduce bureaucratic burdens for researchers and institutions.

In this article, we present evidence of changes in public confidence in science from two sources. First, we synthesize trend data from high-quality survey research organizations. By high quality, we mean research firms that publicly commit to a set of data collection and interpretation practices that increase the likelihood of accurate interpretations. Second, we describe findings from a newly developed approach to analyzing public confidence in science by asking detailed questions not just about science in general but also about perceptions both of scientists’ adherence to the scientific norms they espouse and of the incentives that motivate individual scientists and organizations.

From these data, we conclude that

1. Confidence in science is high relative to nearly all other civic, cultural, and governmental institutions for which data are collected, a conclusion consistent with long-term trends.
2. Confidence has declined…but the decline is not science-specific. Instead, science’s decline is similar to or less pronounced than confidence in many institutions.
3. As of February 2023, the public has high levels of confidence in scientists’ competence, trustworthiness, and honesty. For example, when asked the question “How confident are you that scientists provide the public with trustworthy information about the science in their area of inquiry?” 84% of respondents report that they are very confident or somewhat confident (see *SI Appendix*).
4. However, many U.S. adults question whether scientists share their values and whether they can overcome their biases. For example, when asked, “When a study runs counter to the interests of the organization running the study, which is more likely to happen?” and given a choice between the response “Scientists will publish the finding” and “Scientists will not publish the finding” 70% of the sample chooses the latter (see *SI Appendix*).
5. The public has consistent beliefs about how scientists should act and beliefs that support their confidence in science despite their concerns about scientists’ possible biases and distortive incentives. Eighty-four percent (84%) of U.S. adults responded that it is “somewhat important” or “very important” for scientists to disclose their funders. Ninety-two percent (92%) offered the same responses to a question about the importance of scientists “being open to changing their minds based on new evidence” (see *SI Appendix*).

Collectively, these data clarify how members of the public view science and scientists and reveal details about what types of actions could affect their confidence in science and scientists going forward.

We proceed as follows. First, we describe our data selection criteria. Many surveys contain questions about public confidence in science but not all hew to widely recognized best practices for making representative claims about the U.S. population. Next, we offer an overview of trends in public confidence in science dating back twenty years. To highlight the importance of science confidence trends, we show results from a study that showed how variations in confidence corresponded to U.S. adults’ decisions to take one of the COVID-19 vaccines. Then, we use results from another study that provides more detailed data to reveal a higher level of nuance in public views of science and scientists. In the final section, we use insights from these data to describe steps that researchers and research organizations who are addressing U.S. audiences can take to increase confidence in their practices and findings when such confidence is warranted. Taking these steps may increase public acceptance of their research.

## Data Selection and Attributes

Survey research provides the empirical corpus from which most of this review draws. Surveys asking questions relating to “confidence” in science vary in quality. We restrict our attention to research from survey organizations that collect data to facilitate in-depth research and that follow best practices concerning scientific sampling procedures. For time-trend presentations that characterize levels of trust in science in the United States, we use data from nationally representative surveys of the population from producers that adopt the American Association for Public Opinion Research (AAPOR) Code of Professional Ethics and Practices, who are signatories of AAPOR’s *Transparency Initiative* or who have made equivalent methodological commitments, at the time of data collection (6). In other parts of this article, we cite research that documents correlates of scientific attitudes. These studies examine specific relationships and do not make representative claims about the nation. These supplementary studies also use well-documented and publicly accessible methodologies.

When interpreting survey research findings, the wording of questions matters. For each finding below, we present the exact question wording that elicited the response. Researchers sometimes word questions in different ways. For example, some ask about confidence in “science” while others focus on confidence in “the scientific community” and still others concentrate on “trust.” These variations are a normal part of survey research, reflecting the fact that researchers have diverse interests and measure different phenomena. In many media and other public conversations about confidence in science, there is a focus on “trust in science” and concerns that “trust is falling.” While recognizing that trust appears more frequently in common parlance, the term confidence more accurately reflects the questions that prominent survey research organizations ask. We will say more about what findings about confidence imply about trust in science in the article’s final section.

## Science Confidence Time Trends

**Fig. 1.**
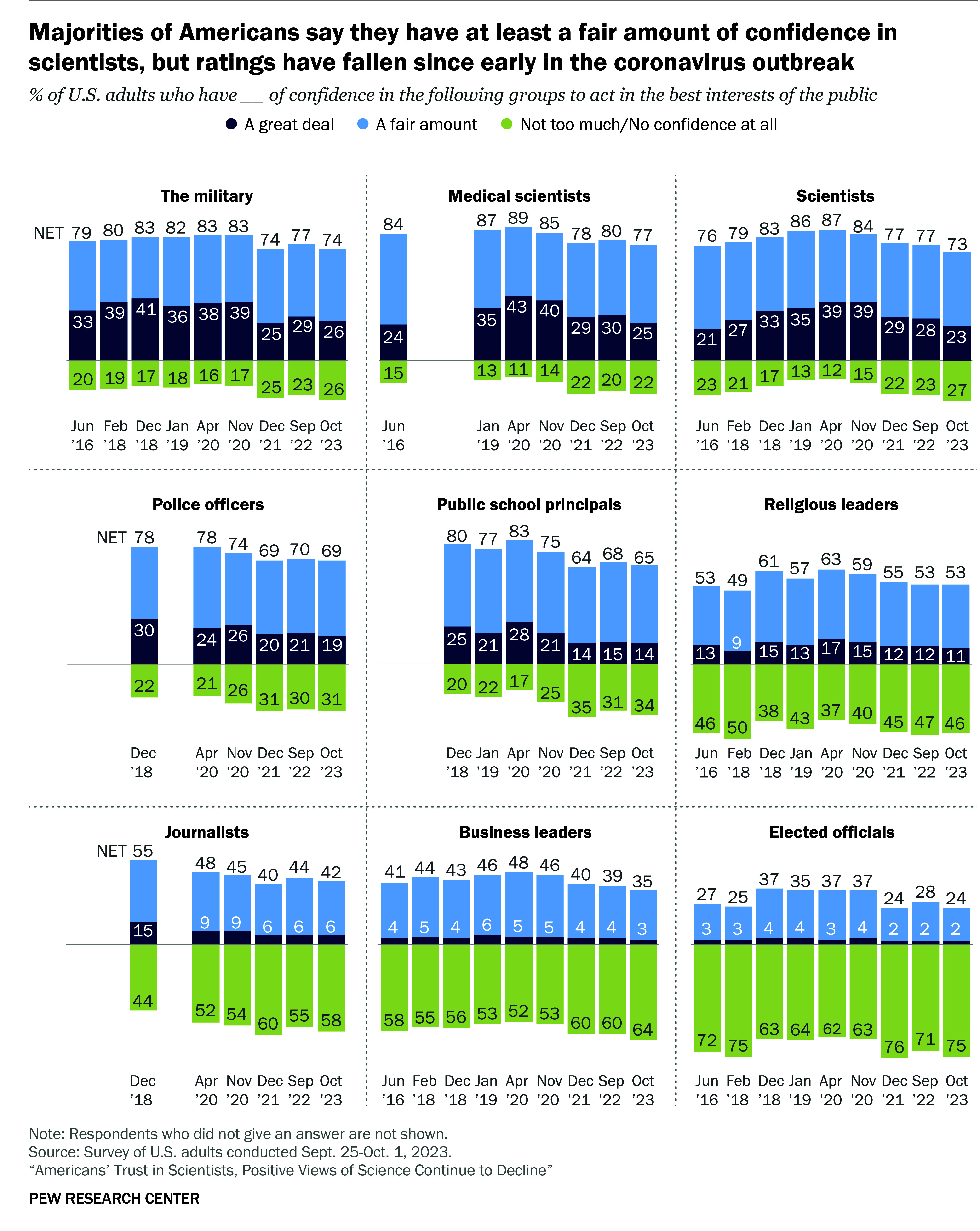
Recent U.S. trends in public confidence in scientists, medical scientists, and other institutions (2).

To measure confidence in U.S. civic institutions, the Pew Research Center asks, “How much confidence, if any, do you have in each of the following to act in the best interests of the public?” For each civic institution, survey participants can select one of the following responses, “a great deal of confidence,” “a fair amount of confidence,” “not too much confidence,” “no confidence at all,” or they can choose not to answer. Pew asks this question about many groups including scientists, medical scientists (Pew is one of only a few survey research organizations that asks these questions separately), the military, police officers, religious leaders, journalists, elected officials, and more. In Fig. 1, we draw from Pew’s recorded confidence changes from 2016 to 2023 (2).

The figure’s *y*-axis refers to the percentage of respondents who answer the question in a specific way. The dark blue parts of each horizontal bar refer to the percentage responding, “a great deal of confidence.” The light blue part represents the percentage responding, “a fair amount of confidence.” The green part refers to the combined percentage who report “not too much” or “no confidence at all” in the named civic institution.

Fig. 1 shows high levels of confidence in “scientists” and “medical scientists” relative to other groups. These levels are nearly identical to confidence in “the military” and higher than that for police officers, religious leaders, journalists, business leaders, and elected officials. These data also show that U.S. public confidence in scientists and medical scientists dropped from 2020 to 2023. This decline, however, is not unique. All measured institutions experienced declines over the same period with most similar in size to the decline in science.

In its General Social Survey, the National Opinion Research Center at the University of Chicago (NORC) studies a similar phenomenon. Where Pew asked about scientific institutions, NORC asks about the individuals running scientific institutions. Specifically, “I am going to name some institutions in this country. As far as the people running these institutions are concerned, would you say you have a great deal of confidence, only some confidence, or hardly any confidence at all in them?” Where Pew asks about confidence in “scientists,” NORC asks about confidence in “the scientific community.” Since our goal is to show apples-to-apples comparisons, we need each organization to ask the question in an internally consistent way over time. The Pew and NORC, for each of their surveys, satisfy this condition.

**Fig. 2.**
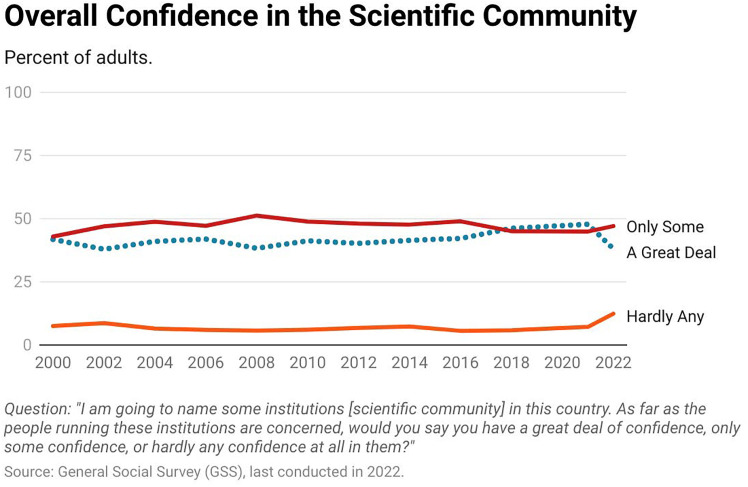
Overall public confidence in the scientific community in the United States (7). Image credit: Ciera Hammond.

Fig. 2 reveals the trend in U.S. public confidence changes from 2000 to 2022 (7). In it, the dotted blue line refers to the percentage responding, “a great deal of confidence,” the solid red line represents the percentage responding, “only some confidence” and the solid orange line refers to the percentage who report “hardly any confidence at all” in the scientific community. Fig. 2 shows that confidence in the people running the scientific community has been high over the last two decades. Even with a sharp decline in 2022, over 85% of U.S. adults report having “a great deal” or “some confidence” in the scientific community every year of the survey. Krause et al. (8) and Brady and Kent (9) find similar trends over different periods using comparable sources of data.

Collectively, the survey data offer evidence that within the United States:

• Confidence in scientists and the leaders of the scientific community is high relative to other groups.
• This confidence has declined in recent years, but the science-related decline is comparable to, or less pronounced than, declines in confidence in other groups.

## Association with COVID-19 Vaccination Status

Confidence in science provides a reason for people to pay attention to scientific findings. This confidence becomes more important when people are asked to weigh scientific evidence. A case in point is the correspondence between people’s views of science and their willingness to take a life-saving vaccine. Survey researchers from several organizations examined this relationship. Each found important relationships within the U.S. adult population between a science-based form of trust and willingness to take a COVID-19 vaccine.

For example, the Jamieson et al. (10) survey empaneled citizens of Florida, Michigan, Ohio, Pennsylvania, and Wisconsin seven times between April 2020 and March 2021. About trust, they asked three questions:

• “How much, if at all, do you trust the leaders of institutions such as the U.S. Centers for Disease Control and Prevention (CDC) and the National Institutes of Health (NIH) to act in the best interest of people like you?”
• “How much, if at all, do you trust what Dr. Anthony Fauci of the National Institutes of Health (NIH) tells you about the coronavirus pandemic?”
• “How much, if at all, do you trust what the U.S. Centers for Disease Control and Prevention (CDC) tells you about the coronavirus pandemic?”

For each question, response options were “a great deal,” “a lot,” “a moderate amount,” “a little,” and “not at all.” They then aggregated responses to these questions to form a “trust in health authorities” measure. About willingness to vaccinate, they asked, “If a no-cost vaccine that protects people from the coronavirus, also known as COVID-19, becomes available and is approved by the Food and Drug Administration, also known as the FDA, how likely, if at all, would you be to get vaccinated?” Response options were “not at all likely to get vaccinated,” “not too likely,” “somewhat likely,” and “very likely to get vaccinated.”

**Fig. 3.**
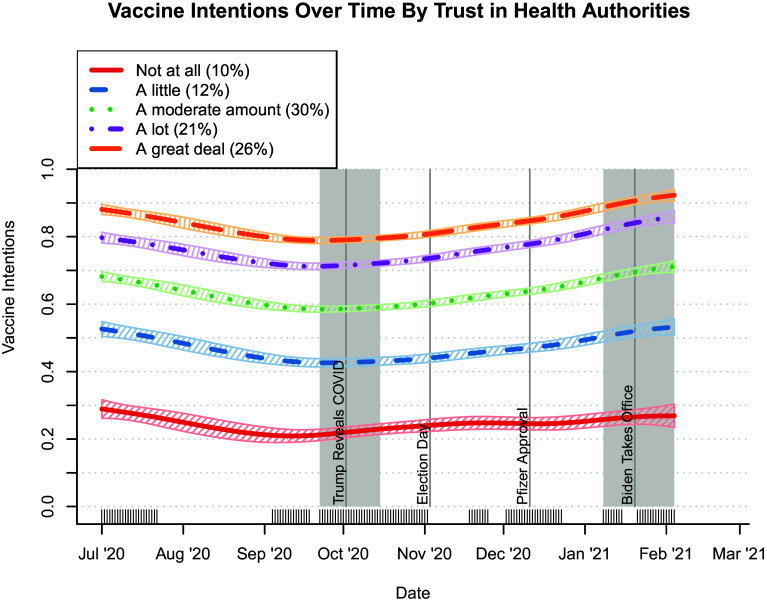
Correspondence between the U.S. adult population’s trust in health authorities and intention to vaccinate. Survey data were collected in different waves from July, August/September, September/October, October/November, November, and December of 2020, and January/February of 2021. The left-hand gray region is the September/October 2020 wave and the right-hand gray region is the January/February 2021 wave (10).

Fig. 3 depicts the relationship between these variables from July 2020 to February 2021. The figure reflects their main finding, which is that the U.S. adult population’s trust in health authorities was a significant predictor of their reported intention to vaccinate. Allington et al. (11) report similar correspondences in the United Kingdom.

In addition, Jamieson et al. (10) and the COVID States Project (12) also found that higher levels of trust in health authorities were strongly and negatively correlated with subsequent acceptance of COVID-related misinformation. In other words, people in the United States with lower levels of trust were more likely to accept misinformation, which, in turn, was associated with a lower reported willingness to vaccinate.

Given the demonstrated effectiveness of COVID-19 vaccination at saving lives, mitigating hospitalization, and reducing many related public health risks, these data offer evidence of how levels in public confidence in science correspond to real quality-of-life outcomes.

Collectively, the research described in this section offers evidence that within the United States:

• Lower levels of trust in the public health institutions and spokespersons that communicate health science were associated with acceptance of misinformation about COVID-19 and COVID-19 vaccines.
• Acceptance of misinformation about COVID-19 vaccines was associated with decisions not to take a life-saving vaccine.
• Higher levels of trust in the public health institutions and spokespersons that communicate health science were associated with taking a COVID-19 vaccine.

## A Closer Look at Underlying Factors

In most cases, when people are asked to consider conditioning their actions or behaviors on a scientific finding, they are not simultaneously offered the underlying data, code, or supplementary materials that researchers would use to evaluate a scientific claim. In many cases, the public is asked to base their acceptance of a scientific claim on trust—trust in methods, processes, people, or institutions. Wintterlin et al. (13) acknowledge the challenge facing those who are thinking about whether to condition their decisions on a scientific finding when they write (pp. 1–2):

Scientists (and science as a whole) provide evidence and advice for societal problem solving and collective decision-making. For this advice to be heard, the public must be willing to trust science, where “trust” means that one can confidently expect science to provide reliable knowledge and evidence… Because of their bounded understanding of science, citizens inevitably must trust in science (or scientists as representatives of that system), even though this might be risky…

To gain a better understanding of why U.S. adults vary in their willingness to take this kind of risk, the Annenberg Public Policy Center surveyed an empaneled nationally representative sample of U.S. adults (see *SI Appendix* for methodological details). This study, called the Annenberg Science Knowledge survey, or ASK, offers a way for researchers to distinguish a range of public views about science and scientists. The ASK survey posed questions not just about science and scientists in general, but also examined whether the public viewed scientists at universities differently than scientists who work for the federal government or in industry. While the ASK survey included an expansive set of variables, we focus here on those that pertain to confidence in science or scientists in general. Data reported below are from an ASK survey conducted between February 22 and February 28, 2023, on a representative sample of 1,638 empaneled U.S. adults.

The survey initially asked, “In general, how confident are you that scientists provide the public with trustworthy information about the science in their area of inquiry?” The response options were “very confident,” “somewhat confident,” “not too confident,” “not confident at all,” and “don’t know.” Thirty-eight percent (38%) reported that they were “very confident.” Forty-six percent (46%) reported being “somewhat confident.” Fourteen percent (14%) reported being “not too confident” and 2% reported being “not confident at all.” Overall, 84% of respondents reported being “somewhat” or “very” confident.

Table 1 shows responses to a more detailed set of questions on how the public perceives scientists. This set of questions began with the instruction, “For each of the following statements about scientists, please indicate whether you agree or disagree with it.” Each subsequent question named a specific characteristic. For many characteristics commonly associated with confidence, the sample reported overwhelmingly positive views of scientists. In February of 2023, over 80% of the sample perceived scientists as “competent,” 70% as “trustworthy,” and roughly 65% as “honest,” “ethical,” and caring about the well-being of others.

**Table 1.** Public perception of scientists in the United States

Scientists in general	% strongly agree	% somewhat agree	Net agree	% neither agree nor disagree	% somewhat disagree	% strongly disagree	Net disagree
Are competent	35	46	**81**	14	3	2	**5**
Are trustworthy	25	45	**70**	21	7	2	**9**
Are honest	22	47	**68**	21	8	3	**10**
Are ethical	23	43	**65**	25	8	2	**10**
Care about the well-being of others	24	44	**68**	24	6	2	**8**
Share my values	11	31	**42**	45	10	3	**13**
Feel superior to others	9	27	**36**	43	15	6	**21**
Are likeable	9	33	**42**	51	1	5	**7**

In some cases, cells do not sum to 100% due to rounding. Bold values indicate a summation of other columns (see *SI Appendix* for methodological details).

On other confidence-related factors, the distribution of responses is different. When asked whether scientists in general “share my values” or are “likable,” less than half the sample agreed with these statements. While very few respondents actively disagreed with the statements, large numbers responded that they “neither agree nor disagree,” signaling less agreement than was observed with the first batch of characteristics.

A subsequent set of questions measured respondent perceptions of science-related outcomes. One such question asked, “Think about science today. In general, do you think science today is more reliable, less reliable, or about as reliable as science was two decades ago?” Fifty-six percent (56%) of respondents chose “more reliable” as opposed to 13% who chose “less reliable.” Thirty-one percent (31%) chose the response “about as reliable.”

Table 2 shows responses to a more detailed set of outcome-related questions. This set of questions began with the instruction “For each of the following statements about science, please indicate whether you agree or disagree with it.” Two questions asked whether findings produced by U.S. scientists have benefited the nation and people like themselves, respectively. Over 70% of the sample agreed with these statements. Another question measured the extent to which respondents agreed or disagreed with the statement “Science creates unanticipated consequences that replace older problems with new problems.” Responses to this question were more ambivalent, with roughly equal numbers of people agreeing, disagreeing, or neither agreeing/disagreeing with the proposition.

**Table 2.** Public perception of science in the United States

Science in general	% strongly agree	% somewhat agree	Net agree	% neither agree nor disagree	% somewhat disagree	% strongly disagree	Net disagree
Scientific findings produced by U.S. scientists in the past decade have benefitted *the country as a whole*	41	39	**80**	12	5	3	**8**
Scientific findings produced by U.S. scientists in the past decade have benefitted *people like me*	38	36	**74**	18	5	3	**8**
Science creates unintended consequences and replaces older problems with new ones	5	32	**36**	35	20	9	**29**

In some cases, cells do not sum to 100% due to rounding. Bold values indicate a summation of other columns (see *SI Appendix* for methodological details).

Other questions focused on public beliefs about actions and motives. As Table 3 shows, many members of the U.S. public have some level of doubt about what scientists and scientific organizations will do when faced with findings that run contrary to a bias or incentive that they might have. Each question in the table begins with the following prompt, “For each of the following statements about scientists, please indicate whether you agree or disagree with it.”

**Table 3.** Public perception of scientists’ bias in the United States

Scientists…	% strongly agree	% somewhat agree	Net agree	% neither agree nor disagree	% somewhat disagree	% strongly disagree	Net disagree
Provide the public with unbiased conclusions about their area of inquiry	12	41	**53**	24	18	5	**22**
Provide the public with unbiased conclusions about the causes of global climate change	22	32	**54**	19	15	13	**28**
Provide the public with unbiased conclusions about the health risks and benefits of COVID-19 vaccines	25	35	**59**	13	16	12	**28**
In general,.., are able to overcome their human and political biases	8	33	**42**	28	23	7	**30**
Do whatever it takes to get grants and publish, even if it means cutting corners	10	31	**41**	32	20	8	**28**

In some cases, cells do not sum to 100% due to rounding. Bold values indicate a summation of other columns (see *SI Appendix* for methodological details).

Table 3 reveals mixed views on how scientists manage their biases generally, and on the specific topics of global climate change and COVID-19 vaccines. In all three circumstances, just over half the sample believed that science or scientists can protect their findings from bias while just roughly a quarter believed that they do not do this (for question wording see Table 3). When asked whether scientists can “overcome their human and political biases” in general, agreement was even lower, with only 8% “strongly agreeing” and 42% agreeing at any level. The final question in this table conveys a related concern. To a question about whether scientists “Do whatever it takes to get grants and publish, even if it means cutting corners”, more respondents agreed with the statement than disagreed with it. Similar concerns are seen in responses to the question “When a study produces a finding that runs counter to the interests of the organization running the study, which is more likely to happen?” The choices were “Scientists will publish the finding” and “Scientists will not publish the finding.” Seventy percent (70%) of the sample believed that scientists *will not* publish the finding. The ASK data show that many U.S. adults are aware of some of the incentive-based challenges and are not certain that scientists, left to their own devices, will take actions that could benefit the public, but go against their own interests.

At the same time, the sample conveys strong views about what the public believes scientists should do in such situations. Table 4 reveals responses to questions that began as follows “When you are deciding whether to believe a scientific finding, how important or unimportant is it to you that the scientists who authored the study….” Then, two best practices were named. One was disclosing funders. The other was “changing their minds based on new evidence.” Although overwhelming agreement is rarely seen in surveys of the U.S. public, answers to these questions reveal high levels of it. Eighty-four percent (84%) of the sample agreed that disclosing funders is important and 92% indicated that changing minds based on evidence is important. In these cases, at least, the U.S. public is very clear about some of the things that it expects science and scientists to do.

**Table 4.** Actions taken by scientists that affect public belief in scientific findings

When you are deciding whether to believe a scientific finding, how important or unimportant is it to you that the scientists who authored the study…	% very important	% somewhat important	Net important	% neither important nor unimportant	% somewhat unimportant	% very unimportant	Net unimportant
Disclose their funders	58	26	**84**	9	3	4	**7**
Are open to changing their minds based on new evidence	66	25	**92**	4	1	2	**4**

In some cases, cells do not sum to 100% due to rounding. Bold values indicate a summation of other columns (see *SI Appendix* for methodological details).

Collectively, this research offers the following evidence:

• An overwhelming majority of U.S. adults perceive scientists as competent, trustworthy, honest, ethical, and as caring about others’ well-being.
• Large majorities believe that research by U.S. scientists benefits people like the respondent and the nation.
• U.S. adults have more mixed opinions about whether scientists share their values, whether scientists protect their work from their personal and political biases, and whether they will follow scientific best practices when doing so goes against their self-interests.
• The public is heavily in favor of scientists disclosing their funders and changing their minds if the evidence justifies doing so.

## Implications for Increasing Public Confidence in Science

To this point, we have suggested that public confidence in science has dropped somewhat, though the decline is not greater than that seen in most other civic institutions. However, many people question the extent to which scientists’ values align with their own and whether scientists and presumably the structures in place protect scientific research from human error and bias. Researchers also have shown an association between lower levels of trust in U.S. health agencies and spokespersons and hesitance to take a life-saving vaccine by the public.

The scientific literature on science communication provides insights into how to engage members of the public in ways that increase the likelihood of engendering confidence. NASEM’s 2017 report *Fostering Integrity in Research* (14) makes clear the connection between the integrity of knowledge and those who perform the research. Specifically (p. 1),

The integrity of knowledge that emerges from research is based on individual and collective adherence to core values of objectivity, honesty, openness, fairness, accountability, and stewardship. Integrity in science means that the organizations in which research is conducted encourage those involved to exemplify these values in every step of the research process.

One constructive way forward is suggested in a key chapter in *The Oxford Handbook of the Science of Science Communication* (15) which extends on this theme. It considers how adherence to these core values should manifest in science communication. In particular, efforts to increase public confidence in science should not be premised on the assumption that society would be better off with higher levels of uncritical trust in the scientific community. Indeed, uncritical trust in science would violate the scientific norm of organized skepticism and be antithetical to science’s culture of challenge, critique, and self-correction [also see Krause et al. (16)]. As a result, a response to concerns about declining confidence in science focused solely on bolstering trust in science would be problematic.

Instead, researchers, scientific organizations, and the scientific community writ large need to redouble their commitment to conduct, communicate, critique, and—when an error is found, or misconduct detected—correct the published record in ways that both merit and earn public confidence. These actions are needed especially with societal and technological changes regularly provoking and amplifying questions about the trustworthiness of scientific activity. The data we have cited suggest that the scientific community’s commitment to core values such as a culture of critique and correction, peer review, acknowledging limitations in data and methods, precise specification of key terms, and faithful accounts of evidence (17) in every step of scientific practice and in every engagement with the public may help sustain confidence in scientific findings. Among the movements serving these ends are those that increase scholarly commitment to reproducibility and replicability in domains of science where those activities help others interpret scientific claims more accurately (18), promote increased transparency in authors’ contributions to scientific publications (19), create greater incentives for open access to important scientific materials (20), increase efforts to expeditiously notify the scientific community of concerns when problems in published work are being investigated (21), ensure that retracted studies are widely known as such (22), and consider ethical implications of fast-moving areas of scientific practice (23).

While critique and self-correction are intrinsic to scientific best practices, attention to public concerns can identify domains for improvement and promote accountability. The inaugural State of Science address, to be hosted by the Strategic Council in June 2024, will serve as a public-facing initiative toward increasing awareness of public concerns and actions to take in response within the scientific community, government funders, and the media. Recognizing that there are several complementary approaches to assessing the state of science, the event will include discussions of a set of reports by groups and agencies that have explored this topic along different lines. This and other efforts to increase scientific integrity—and perceived integrity—can help scientists around the world work in ways that merit and garner greater confidence.
